# Two plus one is almost three: a fast approximation for multi-view deconvolution

**DOI:** 10.1364/BOE.443660

**Published:** 2021-12-07

**Authors:** Manuel Hüpfel, Manuel Fernández Merino, Johannes Bennemann, Masanari Takamiya, Sepand Rastegar, Anja Tursch, Thomas W. Holstein, G. Ulrich Nienhaus

**Affiliations:** 1 Institute of Applied Physics, Karlsruhe Institute of Technology (KIT), Wolfgang-Gaede-Str. 1, 76131 Karlsruhe, Germany; 2 Institute of Biological and Chemical Systems (IBCS), Karlsruhe Institute of Technology (KIT), 76021 Eggenstein-Leopoldshafen, Germany; 3 Centre for Organismal Studies (COS), Universität Heidelberg, 69120 Heidelberg, Germany; 4 Institute of Nanotechnology, Karlsruhe Institute of Technology (KIT), 76021 Eggenstein-Leopoldshafen, Germany; 5 Department of Physics, University of Illinois at Urbana-Champaign, Urbana, IL 61801, USA

## Abstract

Multi-view deconvolution is a powerful image-processing tool for light sheet fluorescence microscopy, providing isotropic resolution and enhancing the image content. However, performing these calculations on large datasets is computationally demanding and time-consuming even on high-end workstations. Especially in long-time measurements on developing animals, huge amounts of image data are acquired. To keep them manageable, redundancies should be removed right after image acquisition. To this end, we report a fast approximation to three-dimensional multi-view deconvolution, denoted 2D+1D multi-view deconvolution, which is able to keep up with the data flow. It first operates on the two dimensions perpendicular and subsequently on the one parallel to the rotation axis, exploiting the rotational symmetry of the point spread function along the rotation axis. We validated our algorithm and evaluated it quantitatively against two-dimensional and three-dimensional multi-view deconvolution using simulated and real image data. 2D+1D multi-view deconvolution takes similar computation time but performs markedly better than the two-dimensional approximation only. Therefore, it will be most useful for image processing in time-critical applications, where the full 3D multi-view deconvolution cannot keep up with the data flow.

## 1. Introduction

Light sheet fluorescence microscopy (LSFM) has become the method of choice for long-term three-dimensional (3D) imaging of developing organisms with high spatial and temporal resolution [1–3]. Its unique orthogonal arrangement of the optical axes of fluorescence excitation and detection causes selective excitation of fluorophores only in the imaging plane [4,5], resulting in low phototoxicity and fluorophore photobleaching as its key advantages over other fluorescence microscopy modalities. Raw LSFM 3D images, however, have two main shortcomings: (1) The resolution along the optical axis of the detection path is (up to three-fold) lower than the lateral resolution, and (2) light in both illumination and detection pathways suffers from scattering and absorption, especially, when imaging deeper layers of the sample (Fig. S1), resulting in low contrast and resolution [6]. Several techniques have been devised to address these issues, including sophisticated hardware-based illumination beam shaping and two-sided illumination [7–10]. Furthermore, image-processing methods have been developed such as deconvolution, multi-view image fusion and multi-view deconvolution (MVD) and applied to LSFM [11–13] and, more recently, also to multiphoton microscopy [14]. For multi-view imaging, samples are typically mounted onto a rotational stage and imaged from different angles swiftly in succession, which ensures that structural changes occurring between exposures are minimized. Although each 3D view displays the same specimen, the information is by no means redundant. For example, a 90° rotation turns poorly resolved components along the axial direction into the image plane, and regions compromised by strong sample scattering and absorption in one view may be much better visible in another. A computer algorithm fuses the individual views, aiming for optimal contrast as well as best possible and isotropic resolution. MVD is a powerful and elegant technique for multi-view fusion within a deconvolution scheme, often based on the Richardson-Lucy algorithm, which takes all views and the associated point spread functions (PSFs) into account [15,16]. Notably, with multiple views of the same object, the ill-posed deconvolution problem becomes more tractable. However, MVD is a challenging, computationally expensive procedure and takes considerable amounts of time even on powerful computers. This poses severe problems to long-term imaging of developing embryos, during which cameras deliver vast amounts of data (up to multiple terabytes per hour) for many days. Thus, there is a great need to process the image data on the fly to remove redundancies from multi-view datasets. Efforts have been made to accelerate MVD processing [17]. For example, one may reduce the dimensionality of the problem by applying MVD only in two dimensions (2D), i.e., in the plane orthogonal to the rotation axis [18]. Indeed, this method greatly reduces computational cost; however, neglecting an entire dimension obviously leads to inferior results.

Here we introduce an approximation to 3D MVD, allowing fast (real-time) processing of large datasets with modest hardware requirements and substantially improved image quality over 2D MVD. Building on the latter approach [18], we have implemented a computationally cheap, one-dimensional (1D) deconvolution of the image along the axis of rotation. Accordingly, we refer to our method as “2D+1D MVD”. To validate this approach, we simulated multi-view acquisition in a light sheet microscope and evaluated the image quality and processing time of 2D, 2D+1D and 3D MVD for quantitative comparison. We further collected data on an imaging phantom consisting of 100-nm fluorescent beads embedded in an agarose gel matrix to analyze the effects of the three MVD methods on image resolution. Finally, we demonstrated the performance of 2D+1D MVD by imaging real biological systems, developing zebrafish (*Danio rerio*) embryos and *Hydra*.

## 2. 2D+1D multi-view deconvolution

**Fig. 1.**
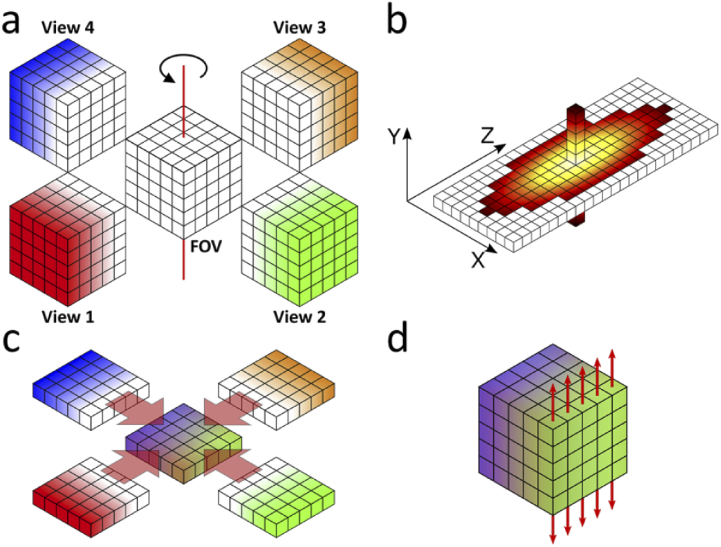
Data processing scheme. (a) Multi-view image acquisition: The region of interest is imaged from multiple angles by stepwise rotation of the sample around the vertical axis (red line). Light emanating from planes close to the camera suffers less from scattering and absorption, so their image quality is better (as indicated by the intensity of the coloring). In each view, the resolution along the optical axis of the detection pathway is lower than the resolution within the lateral image planes. The acquired images are interpolated along the axial direction to achieve an isotropic voxel size. (b) Three-dimensional PSF, approximated by a combination of the 2D horizontal plane and the 1D vertical line through its center. (c) 2D MVD is applied plane by plane in horizontal orientation, resulting in isotropic resolution and a homogeneous intensity distribution. (d) A one-dimensional deconvolution along the rotation axis direction (red arrows) further deblurs the image at low computational cost yet with significant impact on the image quality.

Typical multi-view datasets consist of a small number of views of the same specimen recorded from different angles, which generally differ in image content and resolution (Fig. 1(a)). The chosen number of views is a compromise between image quality, measurement speed, data size and processing time. For example, with only two opposing views, the poor resolution along the optical axis of detection cannot be enhanced in the fusion process. On the other extreme, imaging from many different angles slows processing and does not yield a higher image quality, as the information content becomes increasingly redundant with the number of views. Here, we use four views as a compromise, ensuring that each feature of the sample is captured well in at least two views separated by a 90° rotation. After acquisition, all views are interpolated to isotropic voxel size to prepare them for the ensuing registration step. Precise registration is a crucial pre-processing step to any image fusion approach; it corrects for inaccurate sample positioning and ensures optimal overlap of the individual views. Misregistration inevitably degrades the fusion result by introducing artefacts. We employ a custom-written implementation of an evolutionary search algorithm [19] to find the transformation that ensures optimal overlap of the views. We note that we still critically scrutinize the results of the registration.

After these important pre-processing steps, all views share a common reference coordinate system with isotropic voxel size. For MVD, a 3D PSF is needed that we assume to be constant for all views and their respective fields of view, which in practice may only be an approximation. The key idea of 2D+1D MVD is to approximate this PSF by a 2D PSF in the central plane perpendicular to the rotation axis and an additional 1D PSF along the rotation axis. The latter is given by the intensity distribution of a point-source image along the central line (i.e., the rotation axis) of the 3D PSF. The 2D+1D approximation of the PSF is depicted in Fig. 1(b). Since the MVD is only carried out in two dimensions, the images can be processed slice by slice, enabling a substantial speed-up with respect to 3D MVD.

MVD aims for both isotropic resolution and optimal contrast. Due to varying extents of scattering and absorption along different light propagation pathways through the sample, the image quality of a certain region typically varies between different views. The fusion process should ensure that low-contrast views of a certain sample region should not spoil the content of good ones. To this end, we weigh the information from individual views in each image pixel based on the local entropy, which is an estimate of the local image contrast (see Methods) [20]. As a result, pixel intensities in regions with high contrast contribute more strongly to the fused image. 2D MVD effectively fuses the multiple views with their content into a single, nearly isotropically resolved image (Fig. 1(c)).

In the imaging process, however, the object is actually convolved with a 3D PSF that redistributes intensity also along the third dimension. Consequently, 2D image slices may contain features from neighboring planes. A subsequent, computationally inexpensive 1D deconvolution step, as depicted in Fig. 1(d), corrects for this erroneously distributed intensity and improves the overall MVD result.

## 3. Methods

### 3.1 Multi-view image acquisition

3D multi-view light sheet images were taken on our custom-built, digital scanned laser light sheet microscope (DSLM) with two-sided illumination described in Ref. [21]. In all measurements, two-sided Bessel beam illumination with 488-nm (enhanced green fluorescent protein (EGFP)) and 561-nm (red fluorescent protein (RFP), fluorescent beads) laser light was used. The fluorescence emission was collected with a water immersion objective (CFI-75 LWD 16×/0.8w, Nikon GmbH, Düsseldorf, Germany) and imaged onto a sCMOS camera (ORCAFlash 4.0 V2, C11440-22CU, Hamamatsu Photonics K.K., Hamamatsu, Japan) with a 2048 × 2048 pixel sensor.

3D DSLM image stacks with 400 slices were recorded by collecting images at a rate of 10 s^−1^, 2 × 2 pixel binning and a step size of 2 µm. Each volume (1024 × 1024 × 400 voxels) covers a field of view of 825 × 825 × 800 µm^3^. Immediately after image acquisition, a dark image, which was measured beforehand, was subtracted from each slice of the stack to correct for camera offset. To achieve isotropic voxel size for registration, a bicubic interpolation along the axial direction was performed for all views. We zero-padded the 3D image stacks to cubic shape, as required by our registration algorithm.

For optimal deconvolution, a higher spatial sampling of the emitted fluorescence (smaller voxels) would be desirable. However, our application, live imaging of developing organisms, poses limits in several ways. (1) We need a large field of view (ca. 800 µm in all three dimensions) to capture the entire organism with our camera. (2) Processing the huge amount of data collected in long-time measurements must be manageable by high-performance workstations. We note that the magnification of the microscope is chosen such that the pixel resolution (806 nm) in the image plane is about half the width of the PSF (see below), so that we fulfil the Nyquist theorem. (3) There is obviously a trade-off between pixel resolution and signal-to-noise ratio (SNR). Subdividing the image into smaller pixels requires longer light exposure to achieve the same SNR, which should be avoided in imaging experiments with live organisms to minimize phototoxicity.

### 3.2 Simulation of multi-view image data

We created a ground-truth image based on a synthetic structure with lines emanating from the center in all directions, which we adopted from Ref. [22]. It resembles microtubules in a cell undergoing mitosis (but not to scale with respect to our LSFM voxel sizes). We took only half the original data stack to avoid unnecessarily large datasets, and we scaled and interpolated the volume to 1024 × 1024 × 1024 voxels.

Multi-view image acquisition was simulated by first decreasing the intensity along the optical axis of detection to account for scattering and absorption of the emitted fluorescence (Fig. S2). In principle, the same effect occurs along the excitation optical axis; however, it is in practice negligible due to the two-sided illumination. After this step, the image was convolved with a PSF that we extracted from a measurement of 100-nm fluorescent beads to mimic the anisotropic blurring of an optical microscope. Finally, we added Poissionian and Gaussian noise (with zero mean and variance 1.6 × 10^−5^, using the Matlab function *imnoise*) to include photon and detector noise contributions.

### 3.3 Sample preparation

#### 3.3.1 Fluorescent beads

An imaging phantom was prepared inside a fluorinated ethylene propylene (FEP) tube (diameter 2.34 mm, wall thickness 0.23 mm, Reichelt Chemietechnik GmbH, Heidelberg, Germany), consisting of fluorescent polystyrene beads (F8801, FluoSpheres, Invitrogen, Eugene, OR, excitation/emission peaks 580/605 nm, diameter 100 nm) embedded in an agarose gel, as described in Ref. [21]. Here, however, the gel was kept inside the FEP tube for imaging to ensure equal optical conditions for bead and zebrafish/*Hydra* measurements**.**

#### 3.3.2 Zebrafish embryos

Zebrafish housing and husbandry were performed following the recommendations in Ref. [23]. Experiments were performed with zebrafish embryos of the transgenic line *kca66Tg*, inserted with a histone 2a variant fused with EGFP *Tg(h2az2a:h2az2a-EGFP)* to visualize cell nuclei [24]. Embryos at 8- or 16-cell stage were gently pipetted, with chorion attached, into 1.5% low melting point agarose solution (type VII, A6560, Sigma Aldrich, St. Louis, MO) at 38°C. Subsequently, the solution with the embryos was pulled into a FEP tube (same specifications as above) using a syringe. Once cooled and polymerized, the agarose immobilized the chorion, and the tube containing the sample was introduced into the sample chamber of the microscope from above by mounting it onto a steel rod and inserting it into the rotation stage. Measurements were taken for up to 20 h.

#### 3.3.3 Hydra

We imaged transgenic animals of *Hydra vulgaris*, expressing endodermal EGFP and ectodermal RFP, both driven by an actin promotor. These “reverse watermelon” animals were kindly provided by the laboratory of Prof. Robert Steele (UC Irvine). Polyps were maintained in artificial *Hydra* medium (1 mM CaCl_2_, 0.1 mM MgCl_2_, 0.1 mM KCl, 1 mM NaH_2_CO_3_, pH 6.8) at 18 °C and fed twice or three times per week with freshly hatched *Artemia salina* nauplii. Animals were starved for 48 h prior to the experiments. For LSFM imaging, *Hydra* samples were mounted according to the protocol described for zebrafish embryos.

### 3.4 MVD software implementation

All computations and runtime measurements were performed on a high-end workstation (Intel Core i7-8700K 3.7 GHz CPU with 32 GB RAM) equipped with a Nvidia Quadro RTX 6000 graphics card with 24 GB of memory. Our software also runs on less powerful desktop machines (e.g., Intel Core i7-2600 3.4 GHz CPU with 16 GB RAM; Nvidia GeForce GTX 760 with 2 GB of memory), albeit with longer execution times.

At the heart of our MVD software implementation lies the Richardson-Lucy deconvolution algorithm [15,16],

(1) $$e_{k+1}=\;e_{k}\cdot \left(\frac{i}{e_{k}*f}*\;b\right).$$

Shown here is one iteration, where $e_{k+1}$ and $e_{k}$ are the new and the prior image estimates, respectively, *f* and *b* are the forward and back projector, respectively, i.e., the PSF associated with the image (also referred to as a ‘view’ in MVD analysis), i . The asterisk indicates the convolution operation. Thus, a single iteration involves two computationally extremely demanding convolution operations, which are efficiently carried out as multiplications in Fourier space using fast Fourier transform (FFT) algorithms. For high-speed computation, FFT algorithms are implemented on graphics processing units (GPUs) featuring many cores (e.g., 4608 in the Quadro RTX 6000 graphics card) so that the FFT can be run in a highly parallel fashion. MATLAB (The MathWorks, Natick, MA) is a computing platform that offers several functions (including FFT) that can directly access the parallel computing platform compute unified device architecture (CUDA) by Nvidia. Therefore, we coded the 2D+1D MVD algorithm in MATLAB 2020b. The software consists of two main MATLAB functions, *multiviewdeconv_rl.m* for 2D MVD and *deconv1d.m* for the subsequent 1D deconvolution.

As a preparatory step, the 2D and 1D PSF approximations are extracted as central planes and columns, respectively, from either a set of 3D PSFs (differently shaped PSFs for different views) or a single 3D PSF (assuming the same PSF for all views) using the functions *psf2d.m* and *psf1d.m*. Their PSF outputs are subsequently used in the main functions.

As input data, the 2D MVD routine *multiviewdeconv_rl.m* requires a structure array containing the registered views and an array of 2D PSFs, i.e., one for each view. The number of iterations is a user-defined parameter. In the second convolution of the Richardson-Lucy-algorithm, the back projector, *b*, can be modified, which may lead to faster convergence, as less iterations are necessary [12]. Different modifications have been introduced for MVD and we refer to Ref. [12] for a detailed discussion. In *multiviewdeconv_rl.m*, the user must choose if such an optimization should be applied to the back projector and, if so, which specific kind. For this work, we used the product of all individual optical transfer functions (OTFs) as our compound OTF (“Optimization II”, called “opt2” in our function) for fastest convergence [12]. We also implemented the “Optimization I” variant, which modifies the PSF of each view individually by convolution with the other PSFs. In the *multiviewdeconv_rl.m* function, the user can further select between two methods of weighting the pixels from the different views. After registration, all views share a common coordinate system, but a given pixel has in general a different intensity due to different amounts of scattering and absorption along the different optical paths through the sample. Therefore, in the MVD, the individual views are weighted pixel by pixel using either intensity or local entropy [20,25]. In intensity weighting, the pixel weight of a particular view is simply given by its fractional contribution to the pixel intensity summed up over all views. In entropy weighting, we calculate the local variance, $\sigma^{2}$ , of the intensity of a patch of neighboring pixels. The patch size is a user-defined parameter that should be large enough to obtain good statistics yet small enough to ensure that $\sigma^{2}$ is a local property. The local entropy is calculated according to $H=1/2\ln(2\pi e\sigma^{2})$ , with Euler’s number *e*, using fast GPU computation [25]. The weight of a pixel in a particular view is then taken as its fractional contribution to the local entropy sum over all views. In this work, we always used entropy weighting with a patch size of 11 × 11 pixels. The weights are calculated in the subroutine *weight.m*. The subroutine *binmask.m* produces a 2D binary mask reflecting the image content of the views. It contains ones if at least one view contributes to the image fusion and zeros where all views are zero (due to zero padding prior to registration). The mask is converted to single precision and blurred with a Gaussian kernel (standard deviation 20 pixels) to minimize edge artifacts in the FFT calculations by smoothly reducing intensities and weights of the pixels to zero along the edges. Our routine *multiviewdeconv_rl.m* automatically determines the dimensions of views and PSFs and the available GPU memory. Moreover, it estimates the amount of memory needed for MVD. Accordingly, the views are divided into stacks of slices perpendicular to the rotation axis that can be processed in parallel, thereby ensuring optimal use of the available resources without exceeding the maximum thread size of the GPU. Finally, the MVD is executed. To initiate the iteration procedure, a preliminary estimate of the fusion is produced by averaging over all views. In each iteration, we loop through the individual views and calculate a new estimate according to Eq. (1). Prior to executing the division, zero entries in the image estimate are set to one to exclude division by zero, and the new estimates are calculated pixel by pixel. After completing the selected number of iterations, the multi-view fused 2D image is transferred back from the GPU and returned by the function.

The function *deconv1d.m* carries out the 1D deconvolution in the 2D + 1D MVD algorithm. As input data, it requests a 3D array (i.e., the output of *multiviewdeconv_rl.m*) and a 1D PSF. In addition, the number of iterations for 1D deconvolution have to be specified by the user. Similar to *binmask.m* in 2D MVD, the subroutine *blend1d.m* creates an apodization mask (standard deviation 5 pixels) to reduce edge artifacts. Again, the image is subdivided into blocks for optimal usage of the available GPU memory. These blocks are copied to the GPU one after another, where the 1D deconvolution is performed in parallel. The function returns the deconvolved 3D array.

All MATLAB functions and subroutines are freely available via Github  (https://github.com/NienhausLabKIT/HuepfelM). There, we also provide a standalone software that is controlled via a graphical user interface. It can be run without a MATLAB license and only requires a desktop computer equipped with a Nvidia GPU and sufficient memory.

## 4. Results

### 4.1 Comparison of 2D, 3D and 2D+1D MVD with simulated data

Comparative quality metrics such as the mean squared error (MSE) or the Pearson correlation coefficient (PCC) are powerful tools for a quantitative performance assessment and allow comparison of image processing techniques. However, a ground-truth image is needed as a reference, which is typically not available from an imaging experiment. Therefore, we processed our simulated multi-view dataset (Fig. S2) with the three different fusion methods (2D only, 2D+1D and 3D MVD) and analyzed the image quality and computation time. In the simulated data, there is no positioning error and thus no need for registration, which could introduce uncertainties and complicate a comparison.

**Fig. 2.**
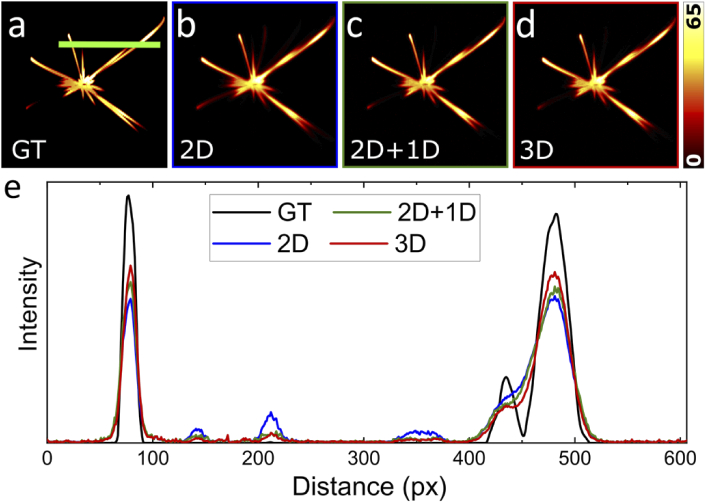
MVD validation with simulated data. (a) Single slice in the XZ-plane of a 3D ground-truth image resembling microtubules in a cell. Results of (b) 2D, (c) 2D+1D and (d) 3D MVD. (e) Intensity across the green line in panel a (averaged over 10 pixels). The black line shows the ground truth (GT). Overall, the 3D MVD (red line) closely tracks the GT. 2D MVD (blue line) shows pronounced crosstalk from neighboring image planes. 2D+1D MVD (green line) redistributes the intensity back to the neighboring planes, yielding a better estimate of the GT.

The three MVD fusions are compared to the ground truth in Fig. 2, showing slices perpendicular to the rotation axis of the simulated 3D dataset (Fig. 2(a)-(d)); results along the rotation axis are displayed in Fig. S3. The benefits of an additional 1D deconvolution are already apparent from visual inspection. For example, in the 2D MVD (Fig. 2(b)), a line is seen extending from the center in the 8 o’clock direction, whereas there is only a localized patch visible in the 2D+1D and 3D MVD images (Fig. 2(c),(d)). Apparently, the line in Fig. 2(b) is due to crosstalk from neighboring planes into the image plane. The additional 1D operation of 2D+1D MVD effectively redistributes image intensity into the planes from where it originated. The line profiles in Fig. 2(e) confirm this conclusion and reveal a close similarity between 2D+1D and 3D MVD.

**Fig. 3.**
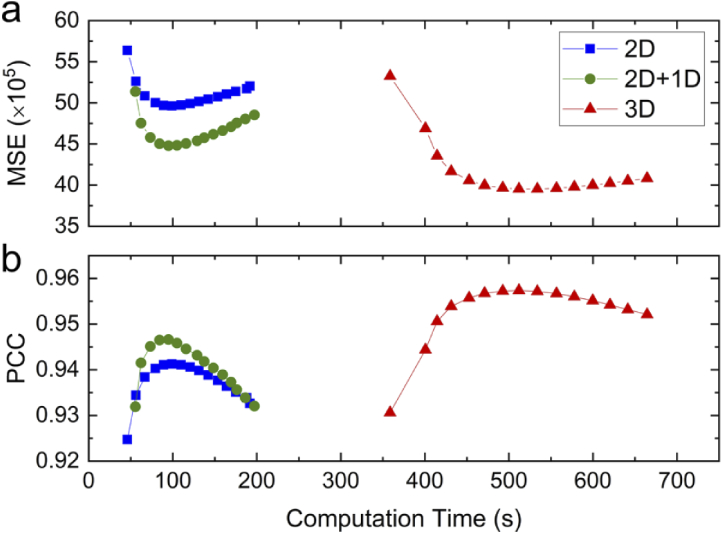
Dependence of the MSE and PCC values on the number of iterations and computation time. (a) MSE and (b) PCC for one to 15 iterations of 2D (blue), 3D (red) and 2D+1D (green) MVD, using the simulated data shown in Fig. 2. Computation time includes the calculation of the weights, with which the different views contribute to the fused image, and data transfer from host to GPU and back. Both 2D and 2D+1D approximative MVD methods are roughly five times faster than 3D processing. Adding a 1D deconvolution does not appreciably increase the processing time. Running too many iterations generates artefacts and again decreases the image quality. Obviously, full 3D processing yields best results. However, the analysis demonstrates that the additional 1D deconvolution substantially improves the image quality at negligible computational cost.

To ensure a fair comparison, we determined the optimum number of iterations of each method by evaluation of the MSE and PCC values, which are plotted for one through 15 iterations against the computation time in Fig. 3. For 2D+1D MVD, three rounds of 1D deconvolution were added after 2D MVD for optimal results (Fig. S4). The data in Fig. 3 clearly prove the enhanced image quality of 2D+1D compared to 2D MVD. Notably, 3D MVD requires more iterations to reach an optimum. We note that the computation times include calculation of the weights with which the different views contribute to the final image and copying data from host to GPU and vice versa. Both approximations, 2D and 2D+1D MVD, run roughly five times faster than 3D MVD. These results imply that 2D+1D MVD offers a good compromise between reconstruction accuracy and computational effort.

In contrast to our findings, Schmid and Huisken [18] previously reported that there is no significant difference between 2D and 3D MVD. For their simulations, they used a small, centered sphere as a ground-truth image. However, the use of such a simple, highly symmetric structure may disguise the disadvantages of the 2D approximation. Moreover, their simulation did not include the intensity attenuation mentioned above, which greatly affects 3D imaging of extended samples and calls for multi-view imaging. It was further claimed in Ref. [18] that 2D MVD enables multi-view fusion in real time by using a streaming approach. To achieve the enormous speed-up, data are loaded from the hard drive, processed and the results are saved in parallel. However, the individual views must already be registered for this approach. For some special LSFM designs, it may be possible to calibrate the microscope before and apply the transformation directly after data acquisition, at least for measurements over shorter periods [18,26,27]. In general, however, this is not the case. As registration also requires reading the data from the hard drive and loading it into the working memory, there is no temporal advantage in saving the registered data and streaming them for MVD processing. In any case, we provide two implementations in our MVD software, one using streaming, i.e., assuming that the data on the hard drive require no prior registration, and another one without streaming, i.e., if the data are already loaded into the working memory, as is the case after registration. Ultimately, the achievable acceleration depends on the enveloping processing pipeline.

We further asked how the 2D+1D approximation performs with respect to 3D MVD if only a single view is acquired. Since fewer data need to be processed, the acceleration must be less. More interesting, however, is the quality of the reconstructed 3D image. How much does it suffer from neglecting one dimension completely, and what can be restored by the additional 1D deconvolution? To address this question, we analyzed a single-view simulated dataset, calculated as described above. We reconstructed the ground truth using the three fusion approaches and evaluated the MSE as a function of computation time (Fig. S5). We found that the 2D and 2D+1D deconvolutions run about three times faster than the 3D deconvolution, rather than five times for MVD. The MSE is reduced by roughly 8% with the additional 1D deconvolution, outperforming the 2D-only approximation. 3D deconvolution, however, reaches closer agreement with the ground truth in a similar amount of time with only two iterations, and more iterations ultimately yield the best results. Therefore, the 2D+1D approximation is only recommendable for processing multi-view datasets.

### 4.2 Application to fluorescent bead images

To evaluate the different fusion methods with real imaging data, we acquired a multi-view dataset on an imaging phantom consisting of sub-diffraction fluorescent beads (diameter 100 nm) embedded in an agarose gel. We took four views separated by 90° angles capturing the same region of interest. After interpolation and zero padding, we obtained a volume of 1024 × 1024 × 1024 voxels with a cubic voxel size with 806 nm edges. We fused the individual images using 2D, 2D+1D and 3D MVD, and analyzed the image resolution along all three dimensions. We also analyzed a single view as a reference for comparison.

To analyze the resolution, we quantified the spatial extensions of the spots corresponding to individual beads as follows. By searching for local intensity maxima in the images, we identified individual beads and determined their center positions. For each bead, we determined the full width at half maximum (FWHM) parameters along the three axes of the intensity distributions. Separately for the three spatial dimensions, we histogrammed the FWHM values from all beads cumulatively and fitted the data by the cumulative normal distribution function to extract the means and their standard errors (Table 1). Unlike the single view data, all MVD fusions provide near-isotropic resolution. Notably, the FWHM along the rotation axis (y-axis) of 2D MVD is larger compared to the single view due to a slight misregistration in the range of one voxel (0.8 µm). 2D+1D MVD resolution parameters are found to be close to those of 3D MVD, attesting to the excellent performance of 2D+1D MVD. Not surprisingly, 3D MVD yields the overall smallest values. Along the rotation axis, 2D+1D MVD outperforms 2D MVD and even yields slightly smaller FWHM values than the single view.

**Table 1.** Mean sizes and their standard errors of the spots from 100-nm beads after deconvolution

Method	FWHM x / µm	FWHM y / µm	FWHM z / µm	# beads
Single View	2.01 ± 0.15	2.07 ± 0.21	9.94 ± 0.50	37
2D MVD	1.99 ± 0.20	2.67 ± 0.12	2.29 ± 0.09	35
2D+1D MVD	1.95 ± 0.16	1.78 ± 0.11	2.26 ± 0.11	36
3D MVD	1.57 ± 0.14	1.48 ± 0.06	2.22 ± 0.17	35

### 4.3 Biological imaging applications

**Fig. 4.**
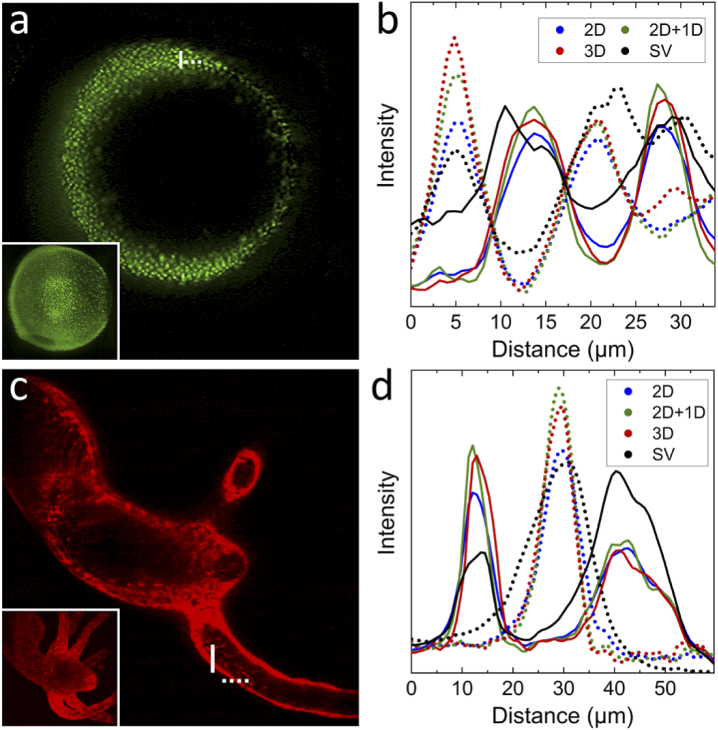
Applications to biological samples. (a) Single slice of a stack showing a developing zebrafish embryo at roughly 90% epiboly (cell nuclei labelled with EGFP) fused with the 2D+1D MVD scheme (five and three iterations for the 2D and 1D deconvolutions, respectively). The inset in the lower left shows a maximum intensity projection of the entire stack. (b) Intensity profiles (averaged over 10 pixels) from 2D, 2D+1D, 3D MVD and a single view (SV) across nuclei (white solid and dashed lines in panel a, corresponding line styles are used for the profiles). (c) *Hydra* expressing RFP in the ectoderm. The 3D image stacks were fused with 2D+1D MVD (five and three iterations for the 2D and 1D deconvolutions, respectively). (d) Intensity profiles (averaged over 10 pixels) from 2D, 2D+1D, 3D MVD and a single view across the ectoderm (white solid and dashed lines in panel c, corresponding line styles are used for the profiles).

Next, we applied MVD to 3D image stacks of live biological specimens (Fig. 4). Fluorescently labeled, transgenic zebrafish embryos expressing histone 2a fused to EGFP, which marks cell nuclei, were imaged during development from four angles separated by 90°. The images were interpolated to isotropic voxel size and zero-padded to cubic shape. After semi-automated, intensity-based registration, the four views were fused using 2D, 2D+1D and 3D MVD. Figure 4(a) displays a slice from 2D+1D MVD. Line profiles through cell nuclei show that the 2D+1D MVD approximation closely tracks the 3D result, whereas there are significant deviations for 2D MVD (Fig. 4(b)). The same effects are visible in Fig. S2 with simulated data along the rotation axis.

In a second experiment, we collected 3D image stacks of *Hydra* expressing EGFP in the inner and RFP in the outer (ectodermal) cell layer. As the animal moves significantly during the experiment, we took only single-color images to speed up data acquisition. The images were processed identically to those of the zebrafish sample. The optimum iteration numbers for MVD determined by visual perception were found to agree with the simulations (Fig. 3): Best results were obtained with five iterations for 2D MVD, three additional iterations of 1D deconvolution for 2D+1D MVD and eight iterations for 3D MVD. A slice from 2D+1D MVD is shown in Fig. 4(c), and the line profiles in Fig. 4(d) again show that 2D+1D MVD follows the 3D MVD result much more closely than 2D MVD. Close-ups of the images processed by the three MVD modalities are depicted in Fig. S6; further images and line profiles along the third dimension are presented in Fig. S7.

## 5. Conclusion

In LSFM, vast amounts of image data are acquired, so data processing can be enormously time-consuming and demands efficient memory management. The problem is further aggravated by multi-view image acquisition, where the amount of data scales with the number of views. Here, we presented a fast approximation to multi-view deconvolution of 3D datasets. Building on 2D MVD, which fuses the image planes of a volume perpendicular to the rotation axis of the multi-view acquisition scheme, we added a 1D deconvolution step along the rotation axis to achieve a closer approximation to full 3D MVD. This additional step significantly improves the quality of the fused image and comes, as we have shown, with negligible expense of computation time. Therefore, this method is attractive for use in time-critical and long-term experiments, where there is a real need to process the images on the fly to squeeze out redundancies and thereby reduce the overall data volume.

## Data Availability

Data underlying the results presented in this paper are not publicly available but may be obtained from the authors upon reasonable request.

## Supplemental document

See Supplement 1 for supporting content.
