# Supplementary material for: Two plus one is almost three: a fast approximation for multi-view deconvolution
Source: Biomed Opt Express. 2021 Dec 7;13(1):147–58. doi: 10.1364/BOE.443660 (PMC8803020; doi:10.1364/BOE.443660)
Supplement: Supplementary file 1 [file boe-13-1-147-s001.pdf]

Supplemental Document

Biomedical Optics EXPRESS

Two plus one is almost three: a fast approximation for multi-view deconvolution: supplement

MANUEL HÜPFEL,[1] MANUEL FERNÁNDEZ MERINO,[1] JOHANNES BENNEMANN,[1] MASANARI TAKAMIYA,[2] SEPAND RASTEGAR,[2] ANJA TURSCH,[3] THOMAS W. HOLSTEIN,[3] AND G. ULRICH NIENHAUS[1,3,4,5,*]

[1]*Institute of Applied Physics, Karlsruhe Institute of Technology (KIT), Wolfgang-Gaede-Str. 1, 76131 Karlsruhe, Germany*
[2]*Institute of Biological and Chemical Systems (IBCS), Karlsruhe Institute of Technology (KIT), 76021 Eggenstein-Leopoldshafen, Germany*
[3]*Centre for Organismal Studies (COS), Universität Heidelberg, 69120 Heidelberg, Germany*
[4]*Institute of Nanotechnology, Karlsruhe Institute of Technology (KIT), 76021 Eggenstein-Leopoldshafen, Germany*
[5]*Department of Physics, University of Illinois at Urbana-Champaign, Urbana, IL 61801, USA*
[*]*uli@uiuc.edu*

This supplement published with Optica Publishing Group on 7 December 2021 by The Authors under the terms of the Creative Commons Attribution 4.0 License in the format provided by the authors and unedited. Further distribution of this work must maintain attribution to the author(s) and the published article's title, journal citation, and DOI.

Supplement DOI: https://doi.org/10.6084/m9.figshare.17096864

Parent Article DOI: https://doi.org/10.1364/BOE.443660

Two plus one is almost three: A fast approximation for multi-view deconvolution: supplemental document

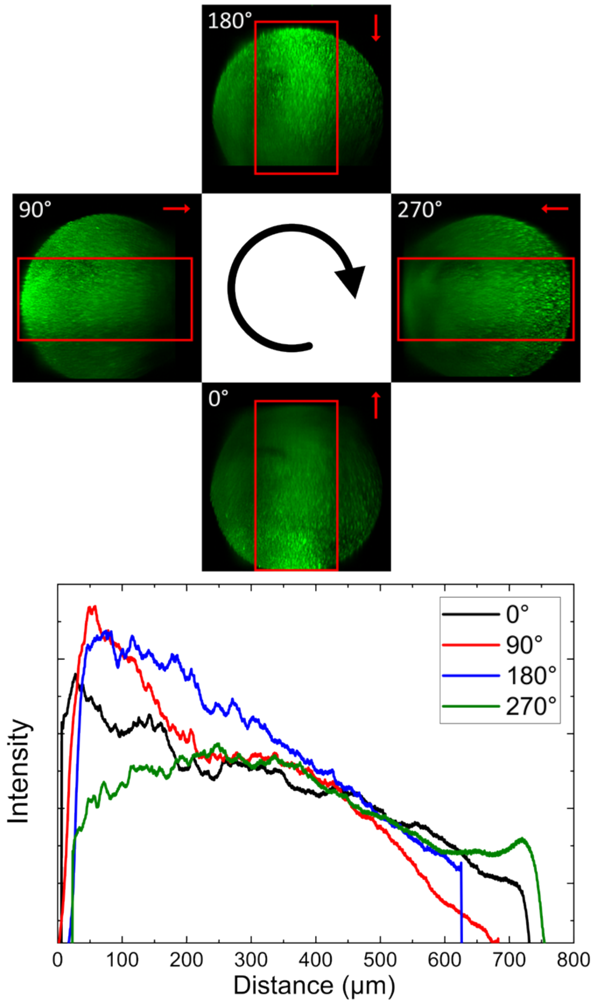

Fig. S1: Intensity attenuation in a multi-view dataset on a developing zebrafish embryo. Shown is the stage of about 100% epiboly, so that the cells form a spherical shell around the yolk. Top: maximum intensity projections (MIPs) along the rotation axis of four individual views. The intensity attenuation is clearly visible from the MIPs themselves. Therefore, the individual views are not redundant but in part complementary in their information content. Bottom: Average intensity within the red boxes as a function of distance from the sample surface; increase in imaging depth is indicated by the red arrows in the images.

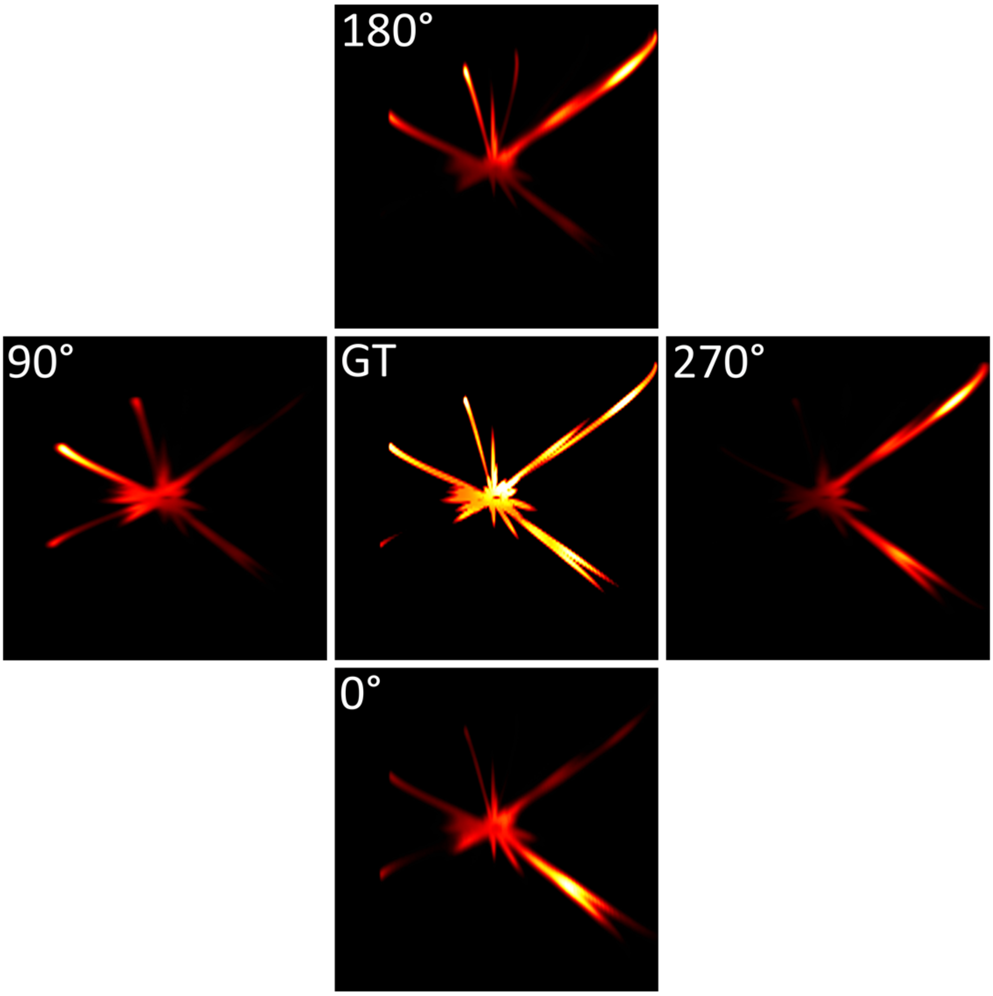

Fig. S2: Simulated multi-view dataset. Shown is a single slice perpendicular to the rotation axis of the ground truth (center) and the four individual views (surrounding). The effects of convolution and intensity attenuation are clearly visible, and it is evident that the individual views are in part complementary in their information content. The attenuation effect with depth in these simulations is analogous to the one seen in the measured data in Fig. S1 (top).

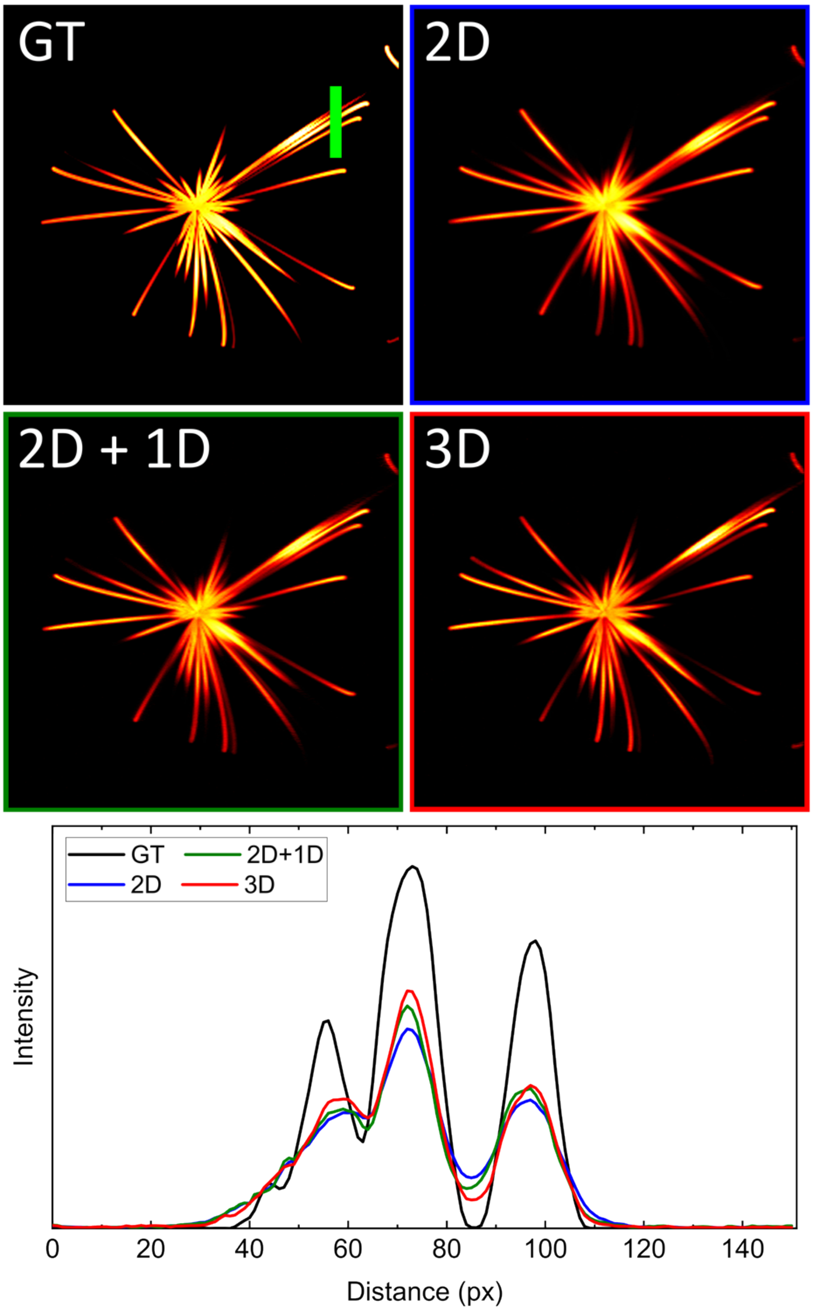

Fig. S3: Line profiles along the 1D deconvolution axis (rotation axis of the sample). Top: Single slice in the X-Y-plane of the ground-truth image and the results of 2D, 2D+1D and 3D MVD. The 1D deconvolution was performed along the vertical (Y) axis. Bottom: Intensity profiles (average over 10 pixels) along the green line (top, upper left) are plotted. The peaks in the 2D MVD profile (blue line) are the broadest; 2D+1D (green line) and 3D (red line) MVD generate narrower peaks.

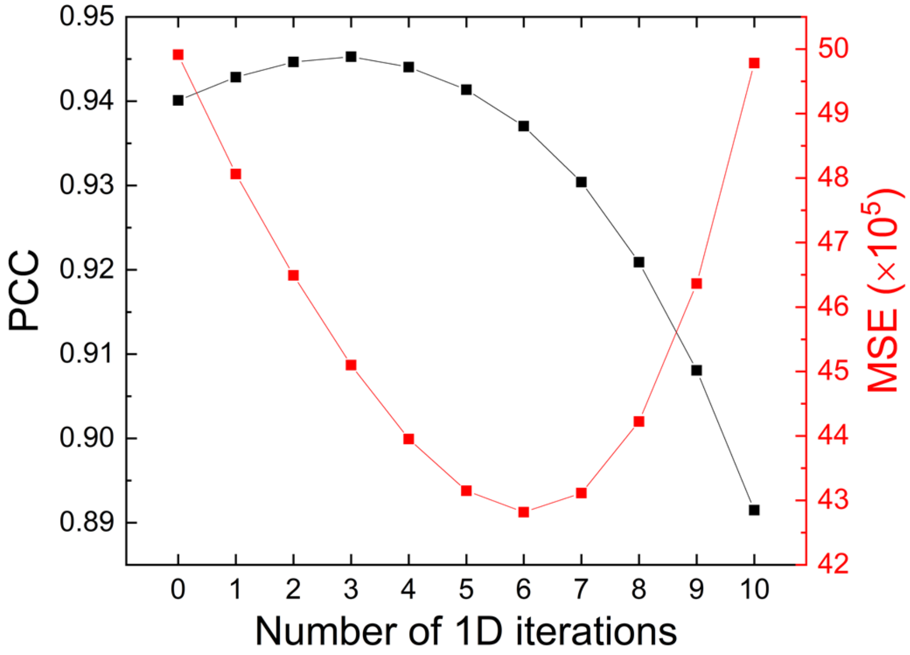

Fig. S4: Pearson correlation coefficient (PCC) and mean-square error (MSE), shown as functions of the number of 1D iterations in the 2D+1D MVD. To determine the optimum number of 1D iterations, we calculated the PCC and MSE after each iteration. PCC and MSE values imply that three and six iterations, respectively, are the best choice. In general, a fewer number of iterations is preferable to avoid artifacts (e.g., noise amplification). Notably, the optimal number of iterations depends on the noise level and should be chosen individually for each dataset.

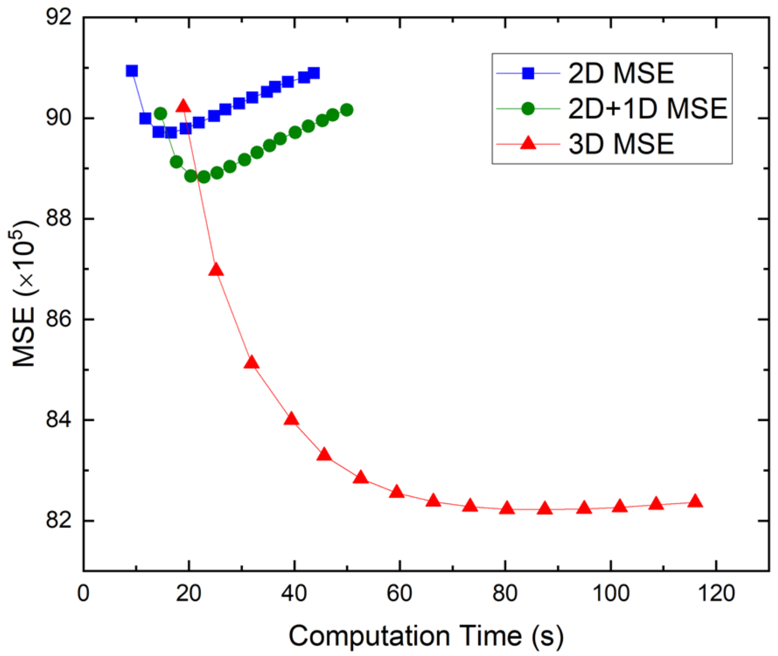

Fig. S5: Mean square error (MSE) of single-view deconvolution as a function of computation time. The MSE determined for one to 15 iterations, using 2D (blue), 2D+1D (green) and 3D (red) single-view deconvolution. 2D and 2D+1D deconvolution reach their minimum roughly 3 times faster than 3D deconvolution. The MSE of 2D+1D deconvolution is clearly smaller than the one for 2D deconvolution only. However, 3D deconvolution ultimately yields much better results than the approximations.

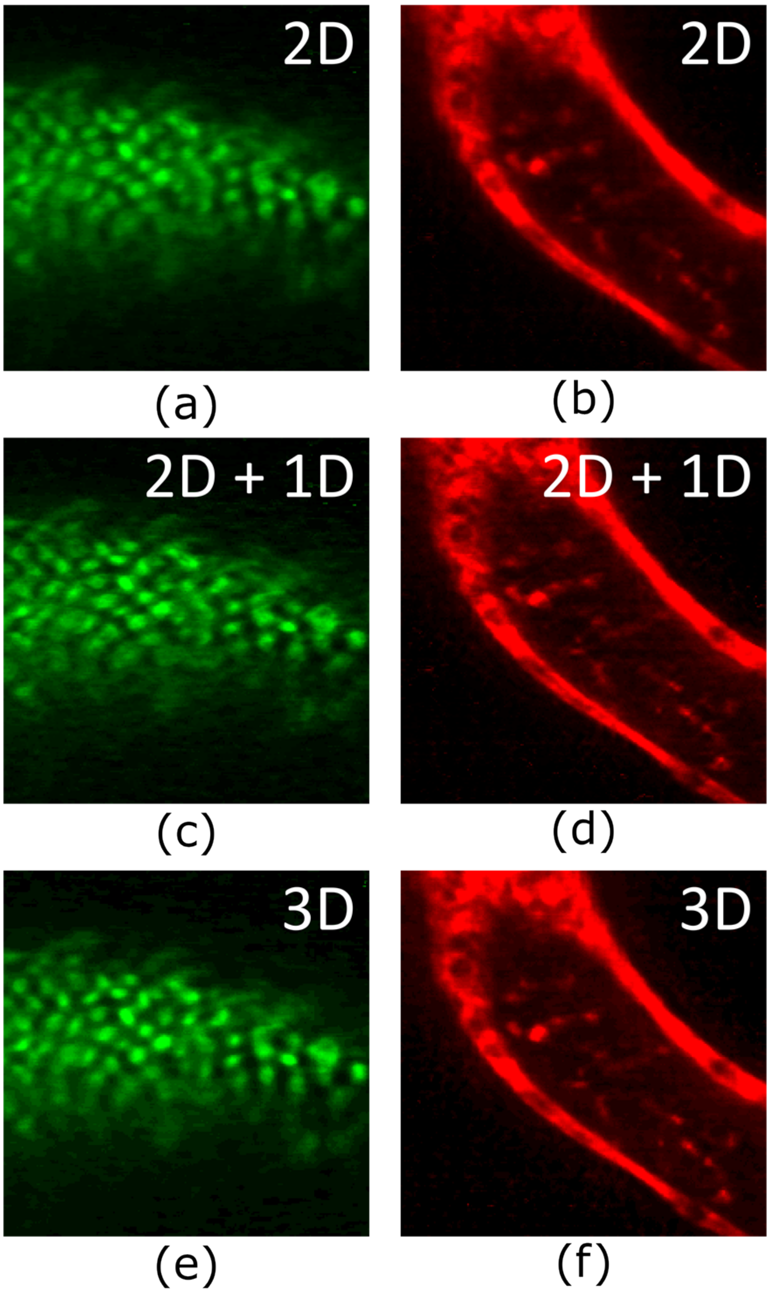

Fig. S6: Close-ups of the images shown in Fig. 4. Zebrafish embryo and hydra images (176 × 176 μm^2) from a 3D dataset processed with (a,b) 2D, (c,d) 2D+1D and (e,f) 3D MVD. Careful inspection reveals that 2D+1D MVD has higher contrast and is more similar to 3D MVD than 2D MVD.

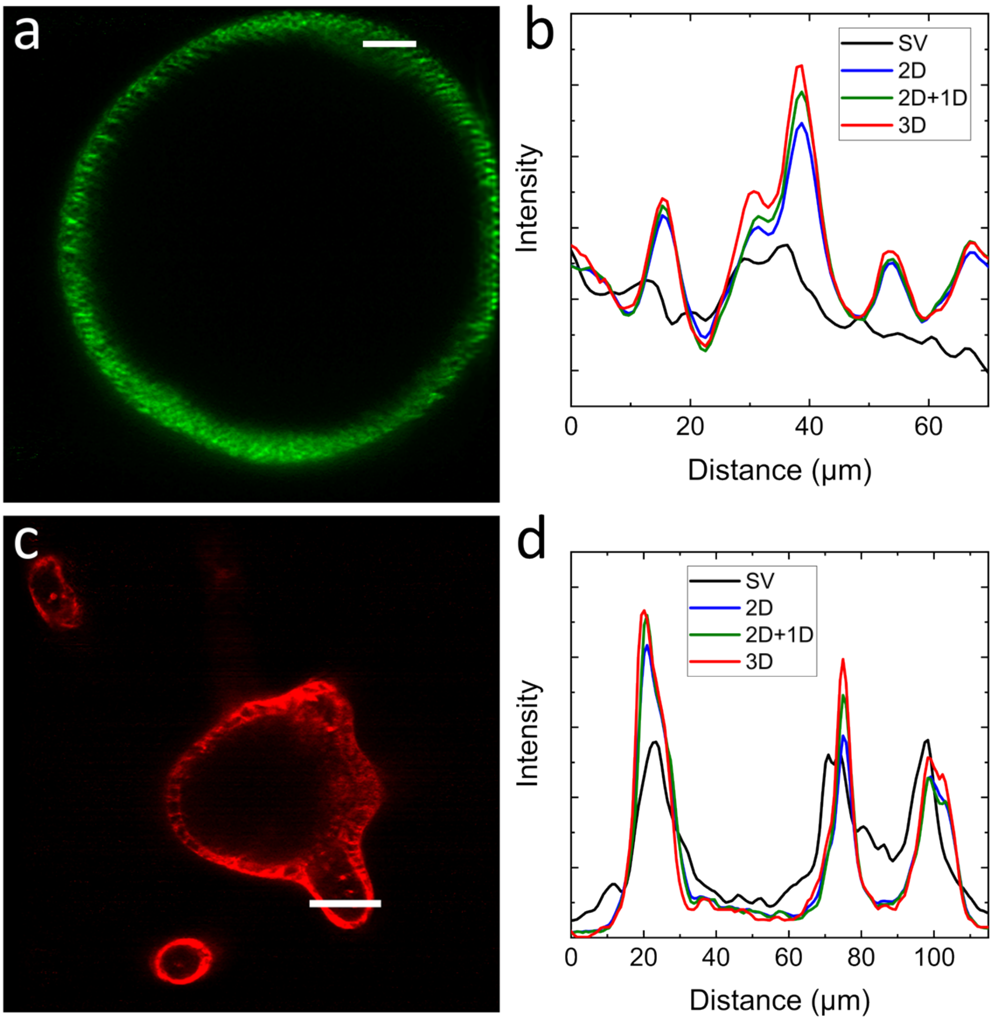

Fig. S7: Applications to biological samples. For the data in Fig. 4, we show images and line profiles along the third dimension. (a) Single slice (perpendicular to the one shown in Fig. 4) of a stack showing a developing zebrafish embryo at roughly 90% epiboly (cell nuclei labelled with EGFP) fused with the 2D+1D MVD scheme. (b) Intensity profiles (averaged over 10 pixels) from 2D, 2D+1D, 3D MVD and a single view (SV) across nuclei (white solid line in panel a). (c) *Hydra* expressing RFP in the ectoderm. The 3D image stacks were fused with 2D+1D MVD. (d) Intensity profiles (averaged over 10 pixels) from 2D, 2D+1D, 3D MVD and a single view (SV) across the ectoderm (white solid line in panel c).
